# Groin Hernia Secondary to Traumatic Pubic Diastasis: An Uncommon Anatomical Sequela

**DOI:** 10.7759/cureus.105032

**Published:** 2026-03-11

**Authors:** Basavaraj Chougala, Shalu Gupta, Amit Goyal, Ashwani Jangir

**Affiliations:** 1 General Surgery, SMS Medical College and Hospital, Jaipur, IND

**Keywords:** groin hernia, mesh repair, orif, pelvic trauma, pubic diastasis, recurrent hernia

## Abstract

Groin hernias are common surgical conditions; however, their occurrence in association with traumatic pubic diastasis is rare and may pose significant diagnostic and operative challenges. Pubic symphysis diastasis alters pelvic biomechanics and disrupts anatomical landmarks, potentially contributing to hernia formation, recurrence, and surgical complexity. We report the case of a 27-year-old male who presented with recurrent left groin swelling and a history of pelvic trauma following a road traffic accident two years earlier. He had previously undergone open mesh hernioplasty and subsequently transabdominal preperitoneal repair for recurrent inguinal hernia. In the current presentation, clinical examination revealed a reducible swelling with cough impulse. Pelvic radiography and 3D CT imaging demonstrated pubic diastasis measuring approximately 6 cm. During surgery, distorted inguinal anatomy and herniation of the urinary bladder and small bowel through the widened pubic symphysis were identified. The herniated contents were reduced, and open reduction and internal fixation of the pubic symphysis were performed, followed by onlay mesh repair. The postoperative course was uneventful, and the patient remained asymptomatic with no recurrence at one-year follow-up. Pelvic instability and anatomical distortion due to pubic diastasis complicate conventional hernia repair and increase recurrence risk. Comprehensive radiological evaluation and tailored surgical planning are essential in such cases. Addressing both pelvic instability and the hernia defect is crucial to achieving durable outcomes. Groin hernia associated with pubic diastasis is an uncommon but clinically significant condition. A high index of suspicion is required in patients with prior pelvic trauma presenting with recurrent or atypical groin swelling. Combined fixation of the pubic symphysis along with mesh hernia repair provides effective and stable reconstruction, minimizing recurrence risk.

## Introduction

Groin hernias are among the most common general surgical conditions, with a reported lifetime risk of approximately 27% in men and 3% in women [1,2]. Inguinal hernias constitute nearly 75% of all groin hernias and typically result from a weakness in the abdominal wall that permits abdominal contents to protrude through the inguinal canal [3].

The association of groin hernias with pubic diastasis is rare but clinically significant. Pubic diastasis, defined as the separation of the pubic symphysis, results in disruption of the anterior pelvic ring and altered biomechanical load transmission across the pelvis, significantly affecting the pelvic stability [4,5]. These anatomical changes may contribute to hernia formation or recurrence by altering pelvic biomechanics and increasing mechanical strain across the inguinal region [6]. Furthermore, the altered anatomical relationships may complicate standard surgical repair techniques and influence operative planning [7]. Although traumatic pubic diastasis has been well described in pelvic trauma literature, its association with recurrent groin hernia is rarely reported. Only a limited number of reports describe herniation occurring secondary to disruption of the anterior pelvic ring, making the clinical relationship largely anecdotal.

This report highlights the potential for inguinal herniation associated with traumatic pubic diastasis and describes a successful surgical approach for managing this rare and challenging condition. It further emphasizes the importance of recognizing pelvic instability as a potential underlying cause of recurrent groin hernia following trauma.

## Case presentation

A 27-year-old male, a non-smoker with normal body habitus and no history of chronic cough, heavy lifting, or other known risk factors for hernia formation, presented to the surgical outpatient department with a complaint of a left groin swelling for 18 months. The patient had sustained multiple injuries to the lower abdomen and left lower limb following a road traffic accident two years earlier. At that time, radiological evaluation revealed a fracture of the shaft of the left femur, for which intramedullary nailing was performed at a tertiary care center. The patient recovered well, and the implant was subsequently removed during follow-up, after which he remained asymptomatic.

Around six months later, he noticed a gradually progressive swelling in the left groin, which was not associated with vomiting, constipation, or bowel obstruction symptoms. He was diagnosed as having an uncomplicated inguinal hernia and underwent open mesh hernioplasty. However, he developed recurrence, for which a transabdominal preperitoneal (TAPP) repair was performed after two months at another center. However, complete operative records from these earlier procedures were not available for review; therefore, specific details regarding the mesh type, operative findings, and recognition of pelvic anatomical abnormalities during the prior surgeries could not be confirmed.

**Figure 1:**
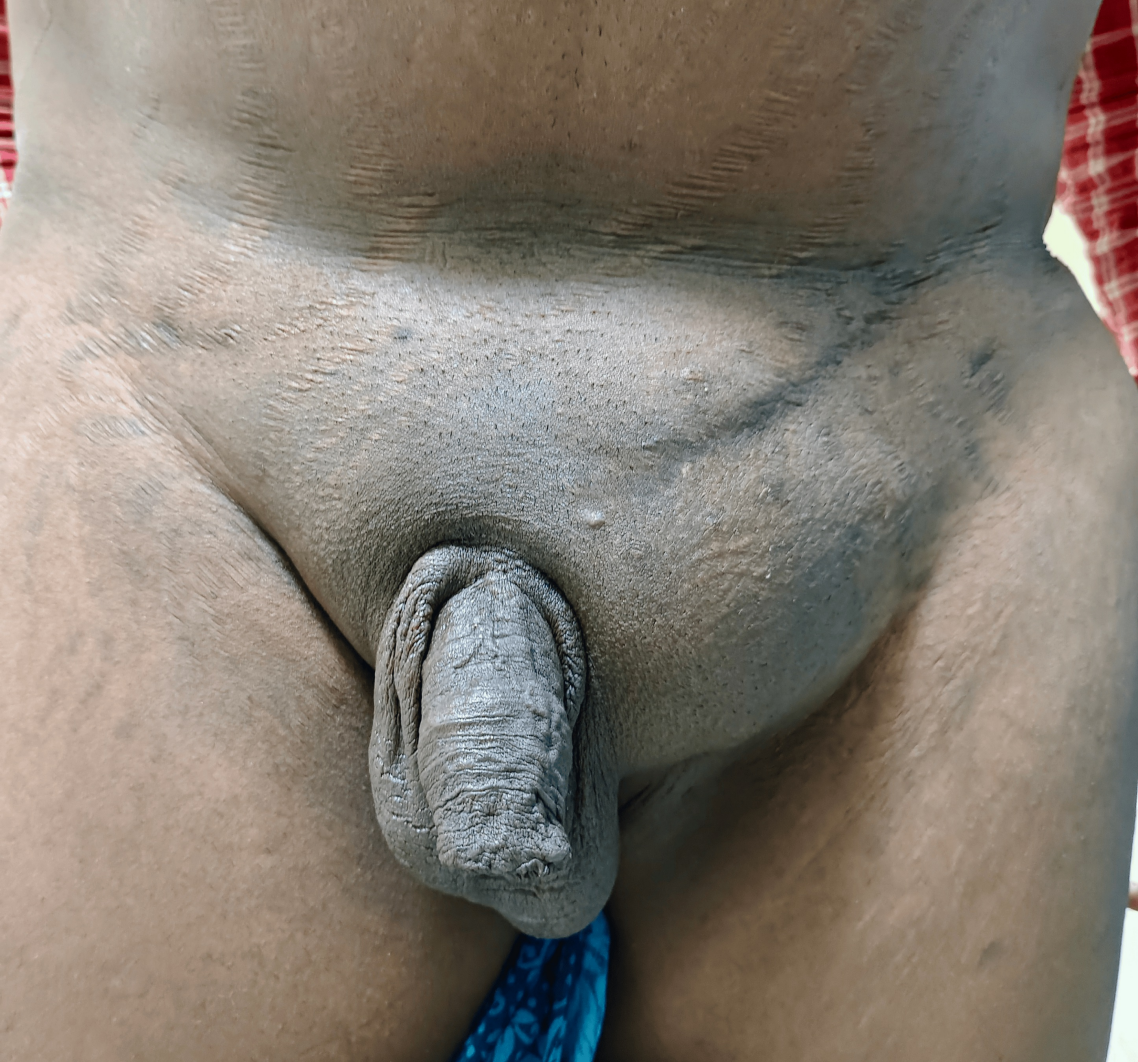
Preoperative clinical photograph showing the left inguinal hernia

**Figure 2:**
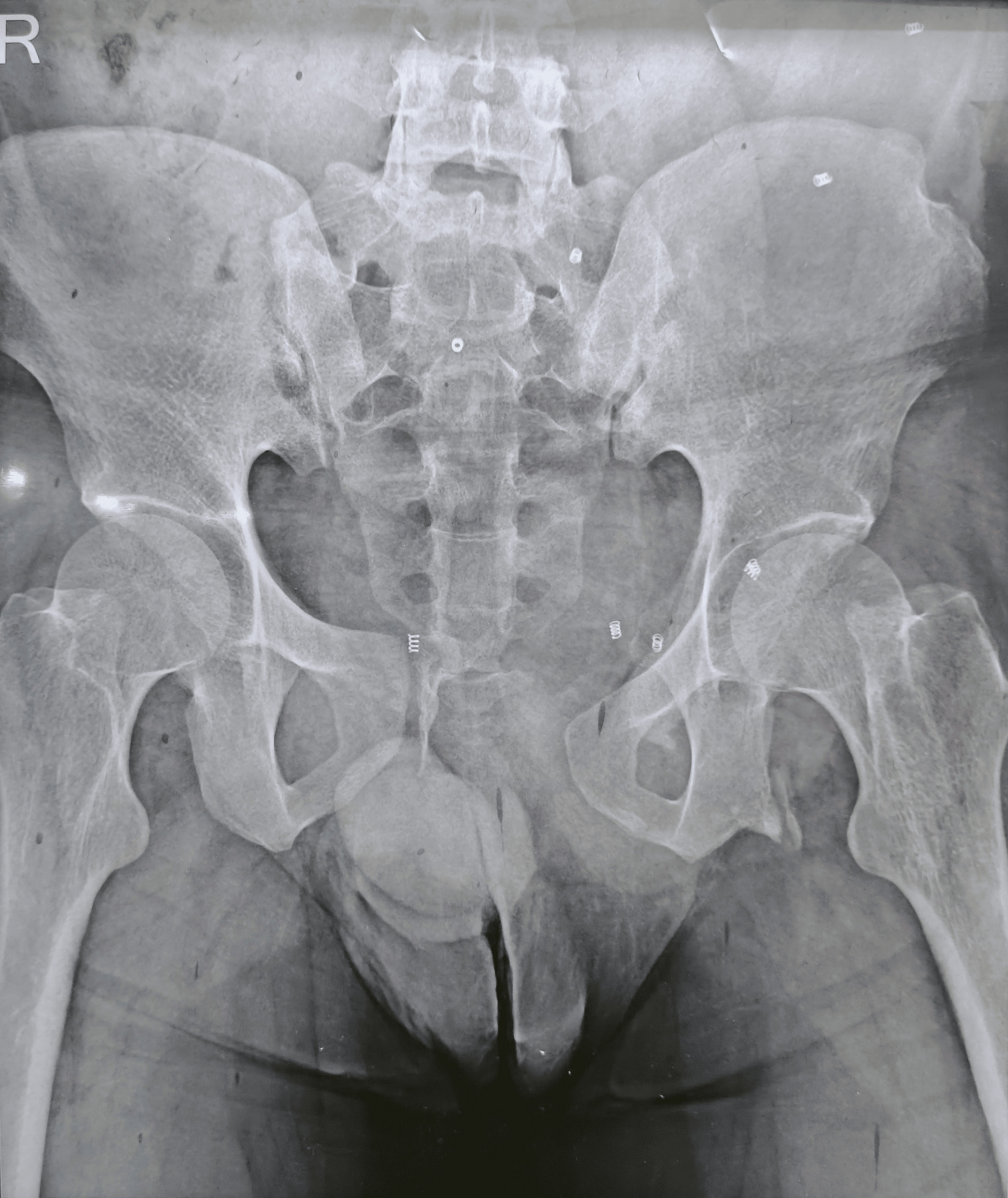
Plain pelvic radiograph demonstrating widening of the pubic symphysis

**Figure 3:**
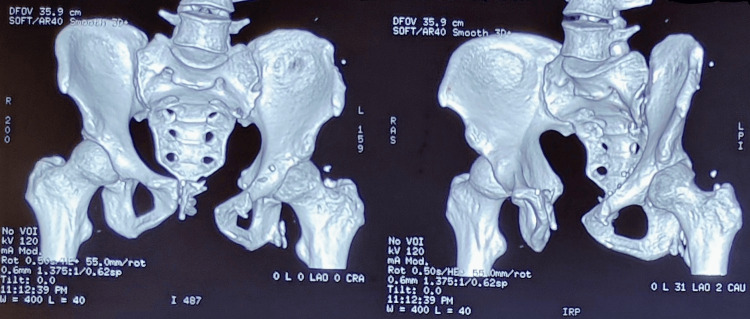
Three-dimensional computed tomography reconstruction of the pelvis revealing pubic diastasis

He now presented again with recurrent swelling in the same region, without pain, any urinary symptoms, or bowel disturbances. On examination, there was a soft, reducible swelling in the left inguinal region extending toward the superficial inguinal ring, with a positive cough impulse. The swelling did not extend into the scrotum, and no features suggestive of femoral or suprapubic hernia were noted. The preoperative clinical appearance is shown in Figure 1. Considering his history of significant pelvic trauma and recurrent hernia, pelvic radiography was obtained to evaluate for possible pelvic ring instability, which revealed pubic diastasis measuring approximately 6 cm. This resulted in significant distortion of the anterior pelvic ring and likely contributed to the failure of the previous hernia repairs. The pelvic radiograph demonstrating pubic diastasis is shown in Figure 2, and the three-dimensional CT reconstruction confirming the widened pubic symphysis is depicted in Figure 3.

At the time of the initial trauma, the detailed pelvic imaging specifically evaluating the pubic symphysis was not performed. Consequently, it remains uncertain whether the pubic diastasis was present immediately following the injury or developed as a chronic sequela of pelvic instability. Additionally, complete radiological records from the time of the initial trauma were not available for review, which represents a limitation in establishing the exact chronology of the pubic symphysis disruption.

Then, the patient was planned for open mesh hernioplasty as there was a history of prior laparoscopic TAPP repair and the anticipated distorted anatomy associated with pubic diastasis, which also allowed simultaneous fixation of the pubic symphysis. Intraoperatively, distorted inguinal anatomy was observed secondary to the widened pubic symphysis. The hernia was identified as a direct inguinal hernia arising through the weakened posterior wall of the inguinal canal in close association with the pubic diastasis. The widened pubic symphysis resulted in disruption of the normal inguinal floor anatomy, through which a portion of the urinary bladder, along with small bowel loops, was found to be herniating.

**Figure 4:**
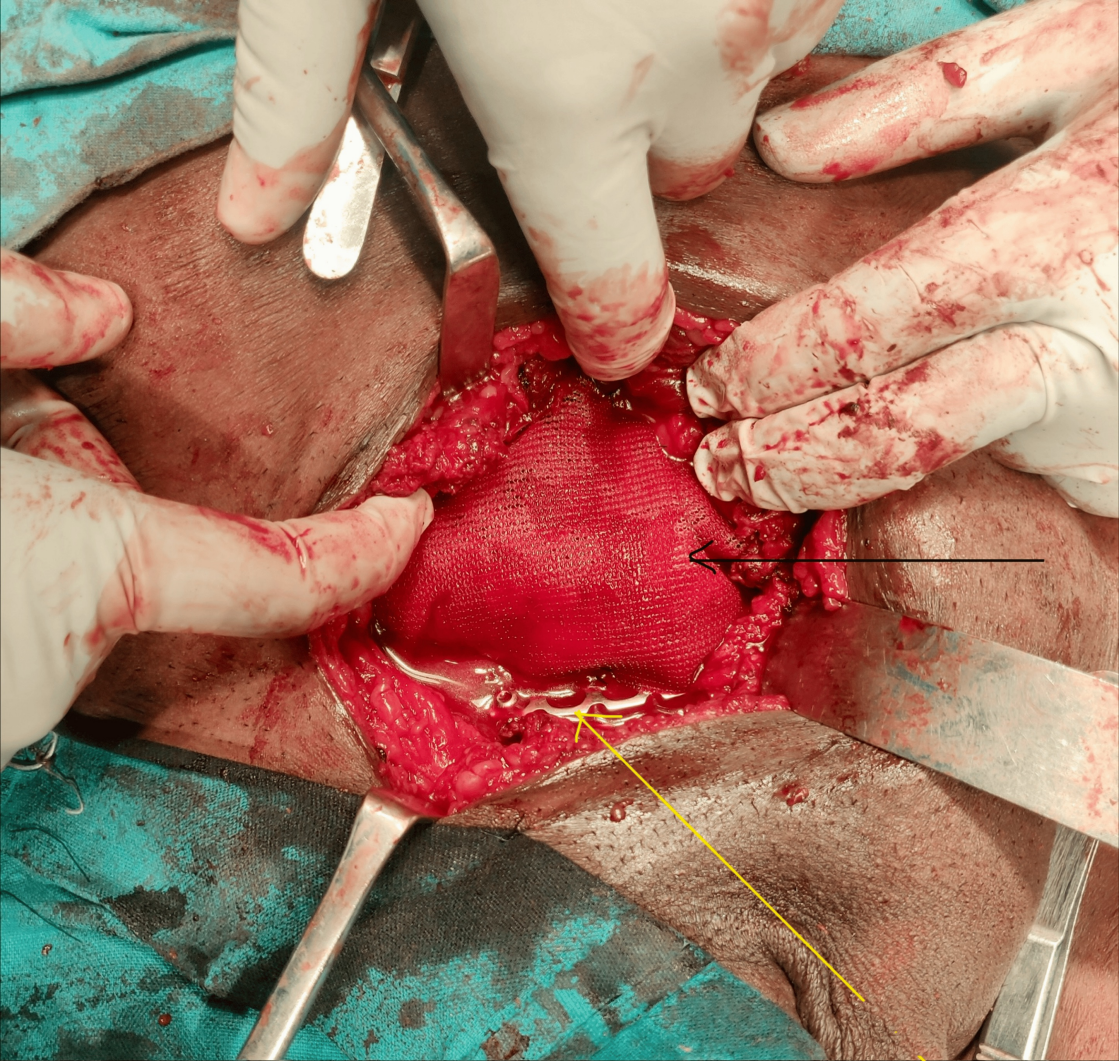
Intraoperative photograph demonstrating plate fixation across the pubic symphysis (yellow arrow) and placement of the polypropylene mesh over the repaired defect in the inguinal region (black arrow)

The herniated contents were carefully reduced. Open reduction and internal fixation of the pubic symphysis were performed using a reconstruction plate with cortical screws placed across the pubic bodies by the orthopedic team. Following restoration of pelvic stability, an onlay polypropylene mesh was placed over the inguinal floor and secured using non-absorbable sutures. The intraoperative findings with mesh placement and plate fixation are illustrated in Figure 4.

The postoperative period was uneventful. At one-year follow-up, the patient remained asymptomatic with no clinical evidence of recurrence. He reported no pelvic pain, urinary symptoms, or functional limitations and had returned to normal daily activity.

## Discussion

This case of a 27-year-old male presenting with recurrent groin hernia in the setting of pubic diastasis highlights the significant challenges encountered when pelvic instability and altered anatomy coexist with a hernia defect. Groin hernias are common; however, their occurrence secondary to traumatic pubic diastasis is uncommon and may easily be overlooked, particularly in patients with prior high-energy pelvic trauma where associated abdominal wall and visceral injuries are common [6,8]. The widening of the pubic symphysis disrupts normal pelvic alignment and biomechanics, compromises the anterior pelvic ring, and weakens the structural support of the lower abdominal wall [4,5]. These changes may predispose to hernia formation and contribute to recurrence following conventional hernia repair.

In the present case, the markedly altered pelvic anatomy made identification of reliable landmarks for safe dissection and mesh placement difficult. The altered anatomy resulting from pubic symphysis diastasis suggests that this presentation represents a complex hernia rather than a routine inguinal hernia. This emphasizes the importance of careful preoperative evaluation, particularly pelvic radiography and CT imaging, in patients with a history of pelvic trauma presenting with groin swelling. Radiological assessment not only assists in defining the extent of pubic diastasis but may also identify associated genitourinary or visceral involvement, which carries significant implications for operative planning and functional outcomes [9,10].

Standard hernia repair techniques may be inadequate in such cases if the underlying pelvic instability is not addressed. Failure to restore pelvic stability may lead to persistent abnormal biomechanical forces across the inguinal region, increasing the likelihood of recurrence and potential mesh-related mechanical failure if underlying instability is not corrected [7,11]. In this context, the inguinal hernia may represent a secondary manifestation of the underlying pubic symphysis diastasis rather than a primary abdominal wall defect. In this patient, a combined approach involving open reduction and internal fixation of the pubic symphysis, along with mesh hernioplasty, provided a stable anatomical reconstruction and resulted in a favorable outcome, with no recurrence during follow-up. Stabilization of the symphysis corrected the primary pathology and restored pelvic anatomy, thereby allowing secure placement of mesh and potentially reducing the risk of recurrence.

This case reinforces the need for a high index of suspicion for pelvic pathology in patients presenting with recurrent or atypical groin hernias, particularly following trauma. In such patients presenting with recurrent groin swelling after pelvic trauma, pelvic radiography or CT imaging should be considered to evaluate for possible pubic symphysis disruption. While previous reports have described traumatic abdominal wall hernias and genitourinary injuries associated with pelvic fractures, herniation secondary to pubic symphysis diastasis remains rarely reported. This case highlights the importance of considering pelvic ring instability as a potential contributing factor in recurrent groin hernias following trauma. Accordingly, management may require a multidisciplinary approach involving both general and orthopedic surgical teams to address the underlying pelvic instability and achieve effective and durable hernia repair. Multidisciplinary evaluation and individualized surgical strategies tailored to the altered pelvic anatomy are essential for achieving durable repair and minimizing the risk of recurrence.

## Conclusions

Inguinal hernia occurring in association with traumatic pubic diastasis is an uncommon but clinically important sequela of pelvic ring disruption. Altered pelvic biomechanics and distorted anatomical landmarks may predispose to hernia formation and contribute to recurrence following conventional repair. In patients presenting with recurrent groin hernia and a history of pelvic trauma, pubic diastasis should be considered as a potential underlying cause, identified preoperatively, and managed accordingly. Thorough radiological evaluation is essential for accurate diagnosis and operative planning. Addressing both the hernia defect and the underlying pelvic instability is crucial to achieving durable repair. Combined fixation of the pubic symphysis with mesh hernia repair may provide an effective treatment strategy in selected cases. Recognition of this rare association is important for surgeons managing recurrent groin hernias in patients with previous pelvic trauma.
